# Decreased methylglyoxal-mediated protein glycation in the healthy aging mouse model of ectopic expression of UCP1 in skeletal muscle

**DOI:** 10.1016/j.redox.2022.102574

**Published:** 2022-12-06

**Authors:** Jinit Masania, Patrick Wijten, Susanne Keipert, Mario Ost, Susanne Klaus, Naila Rabbani, Paul J. Thornalley

**Affiliations:** a Clinical Sciences Research Laboratories, Warwick Medical School, University of Warwick, University Hospital, Coventry, CV2 2DX, UK; b Diabetes Research Center, Qatar Biomedical Research Institute, Hamad Bin Khalifa University, Qatar Foundation, P.O. Box 34110, Doha, Qatar; c Department of Physiology of Energy Metabolism, German Institute of Human Nutrition, Potsdam-Rehbruecke, 14558, Nuthetal, Germany; d University of Potsdam, Institute of Nutrition Science, Potsdam-Rehbruecke, 14558, Nuthetal, Germany; e Department of Basic Medical Science, College of Medicine, QU Health, Qatar University, P.O. Box 2713, Doha, Qatar

**Keywords:** Aging, Uncoupling protein 1, Skeletal muscle, Protein glycation, Methylglyoxal, Proteomics, AGE, Advanced glycation endproducts, AASA, Aminoadipic semialdehyde, CEL, N_ε_(1-Carboxyethyl)lysine, CMA, N_ω_-Carboxymethylarginine, CML, N_ε_-Carboxymethyl-lysine, 3-DG, 3-Deoxyglucosone, 3DG-H, 3-Deoxylglucosone-derived hydroimidazolone, DTNB, 5,5'-Dithiobis(2-nitrobenzoic acid), eIF2α, Eukaryotic initiation factor-2α, FL, N_ε_-Fructosyl-lysine, GSA, Glutamic semialdehyde, Glo1, Glyoxalase 1, GSH, Reduced glutathione, GSSG, Oxidized glutathione, IRE1α, Inositol requiring enzyme-1α, HSA-mUCP1, Mice with UCP1 expression under control of the human skeletal actin promoter, LC-MS/MS, Liquid chromatography-tandem mass spectrometry, MG, Methylglyoxal, MRM, Multiple reaction monitoring, 3-NT, 3-Nitrotyrosine, PENT, Pentosidine, UCP1, Uncoupling protein-1, UPR, Unfolded protein response, WT, Wildtype

## Abstract

Mice with ectopic expression of uncoupling protein-1 (UCP1) in skeletal muscle exhibit a healthy aging phenotype with increased longevity and resistance to impaired metabolic health. This may be achieved by decreasing protein glycation by the reactive metabolite, methylglyoxal (MG). We investigated protein glycation and oxidative damage in skeletal muscle of mice with UCP1 expression under control of the human skeletal actin promoter (HSA-mUCP1) at age 12 weeks (young) and 70 weeks (aged). We found both young and aged HSA-mUCP1 mice had decreased advanced glycation endproducts (AGEs) formed from MG, lysine-derived N_ε_(1-carboxyethyl)lysine (CEL) and arginine-derived hydroimidazolone, MG-H1, whereas protein glycation by glucose forming N_ε_-fructosyl-lysine (FL) was increased *ca.* 2-fold, compared to wildtype controls. There were related increases in FL-linked AGEs, N_ε_-carboxymethyl-lysine (CML) and 3-deoxylglucosone-derived hydroimidazolone 3DG-H, and minor changes in protein oxidative and nitration adducts. In aged HSA-mUCP1 mice, urinary MG-derived AGEs/FL ratio was decreased *ca.* 60% whereas there was no change in CML/FL ratio – a marker of oxidative damage. This suggests that, normalized for glycemic status, aged HSA-mUCP1 mice had a lower flux of whole body MG-derived AGE exposure compared to wildtype controls. Proteomics analysis of skeletal muscle revealed a shift to increased heat shock proteins and mechanoprotection and repair in HSA-mUCP1 mice. Decreased MG-derived AGE protein content in skeletal muscle of aged HSA-mUCP1 mice is therefore likely produced by increased proteolysis of MG-modified proteins and increased proteostasis surveillance of the skeletal muscle proteome. From this and previous transcriptomic studies, signaling involved in enhanced removal of MG-modified protein is likely increased HSPB1-directed HUWE1 ubiquitination through eIF2α-mediated, ATF5-induced increased expression of HSPB1. Decreased whole body exposure to MG-derived AGEs may be linked to increased weight specific physical activity of HSA-mUCP1 mice. Decreased formation and increased clearance of MG-derived AGEs may be associated with healthy aging in the HSA-mUCP1 mouse.

## Graphical abstract

**Figure:**
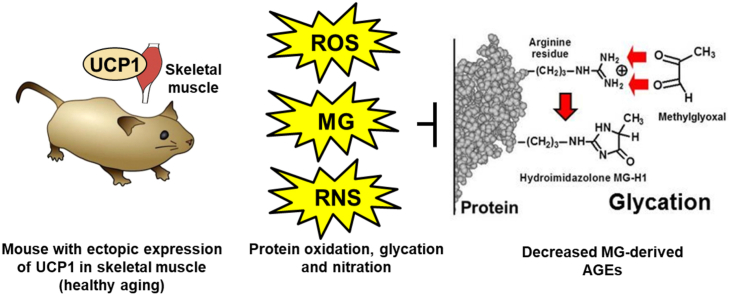

## 1 Introduction

There is an increasing need to understand key metabolic determinants of healthy aging with a view to how physical activity, diet and dietary supplements may be optimized to achieve this. Mouse transgenic and gene knockout models provide key experimental evidence on how changes in gene expression – overexpression or deletion – may have an impact on longevity and sustain healthy aging [1]. One such model has ectopic expression of uncoupling protein-1 (UCP1) in skeletal muscle. UCP1 expression is normally restricted to brown fat mitochondria where it mediates cold-induced non-shivering thermogenesis [2]. Expression of UCP1 in skeletal muscle was achieved under the control of the human skeletal actin promoter (HSA-mUCP1 mice) [3] or rat myosin light-chain 2 promoter (RMYL2-mUCP1 mice) [4]. When expressed in muscle, UCP1 exhibited its usual function of increased uncoupling of mitochondrial respiration activated by fatty acids, with the expression and function of UCP1 in brown fat unaffected by the transgene [5]. In longevity studies, HSA-mUCP1 mice had increased median and maximum lifespan, compared to wildtype (WT) mice on a standard diet [6,7]. The survival advantage was greater on a high calorific diet: median lifespan of HSA-mUCP1 mice was increased 42% on a high carbohydrate, high fat diet where development of obesity was delayed [7]. HSA-mUCP1 mice had lower lean body mass and increased energy expenditure and insulin sensitivity, compared to WT controls [3,4]. Insulin resistance correlated negatively and weight-specific energy expenditure correlated positively with longevity [7].

Metabolically, mice with ectopic expression of UCP1 in skeletal muscle had lower fasting serum glucose and insulin levels, compared to WT controls [3,8]. There was increased basal and insulin-stimulated uptake of glucose by skeletal muscle [9,10] – with increased GLUT4 protein levels and total hexokinase activity [10], and increased whole body glucose metabolism in transgenic (Tg) mice, compared to WT controls, but similar fasting hepatic glucose production rates [9,10]. Transcriptomic studies indicated increased expression of enzymes of amino acid metabolism and antioxidant defense and increased protein turnover in skeletal muscle [11]. Mitochondrial superoxide formation was markedly decreased (*ca.* 76% lower) in Tg mice, likely due to the uncoupling activity of UCP1 [5]. Most prominently, Tg mice show mitochondrial distress–induced skeletal muscle adaptations affecting mainly fast/glycolytic muscles such as quadriceps, which displayed decreased mass and fiber size concomitant with a transition from fast/glycolytic type II fibers towards slow/oxidative type I fibers. For example, at 20 weeks of age, HSA-mUCP1 mice had muscle mass decreased in extensor digitorum longus and quadriceps by *ca.* 40–50% [11]. Characteristics of the HSA-mUCP1 mouse have been recently reviewed [12].

Healthy aging may be associated with decreased accumulation of spontaneous modification of proteins – particularly by processes of glycation, oxidation and nitration. Such spontaneous modifications are usually of low level but may impair protein folding and function. The major types of protein glycation are: modification on the N-terminus and lysine side chains by glucose to form fructosamine adducts – the latter forming N_ε_-fructosyl-lysine (FL) [13]. Other glycation adducts are advanced glycation endproducts (AGEs) - formed by the degradation of fructosamines and protein glycation by reactive dicarbonyl metabolites such as glyoxal, methylglyoxal (MG) and 3-deoxyglucosone (3-DG). Glyoxal is formed by lipid peroxidation, MG mainly by the trace level degradation of glycolytic intermediates, glyceraldehyde-3-phosphate and dihydroxyacetonephosphate, 3-DG by the enzymatic repair and degradation of FL residues, and other process. Major AGEs are: MG-derived hydroimidazolone MG-H1 and FL-derived N_ε_-carboxymethyl-lysine (CML) [14,15]. Pentosidine (PENT), a widely-studied trace-level fluorescent AGE formed mainly from pentose sugar precursors [16], is a protein crosslink related to activity of the pentosephosphate pathway (PPP) [17,18]. Protein oxidation occurs on susceptible amino acid residues – such as cysteine, methionine, tyrosine, tryptophan and others - by reaction with reactive oxygen species (ROS). A further type of protein oxidation is the formation of so-called “protein carbonyls”, α-aminoadipic semialdehyde (AASA) and glutamic semialdehyde (GSA), formed by the oxidation of lysine, arginine and proline residues. Protein nitration occurs by the reaction of proteins with peroxynitrite and other reactive nitrating species. A major protein nitration adduct is 3-nitrotyrosine (3-NT) [19,20]. These protein modifications may be robustly quantified concurrently by stable isotopic dilution analysis liquid chromatography-tandem mass spectrometry (LC-MS/MS). The procedures involved and physiological significance of protein glycation, oxidation and nitration adducts has recently been described [20,21]. Proteome integrity with low, tolerable levels of these damaging modifications is maintained by activation of chaperones to prevent abnormal protein aggregation and to direct ubiquitin ligases to damaged proteins for ubiquitination and proteasomal degradation [[22], [23], [24]].

**Fig. 1:**
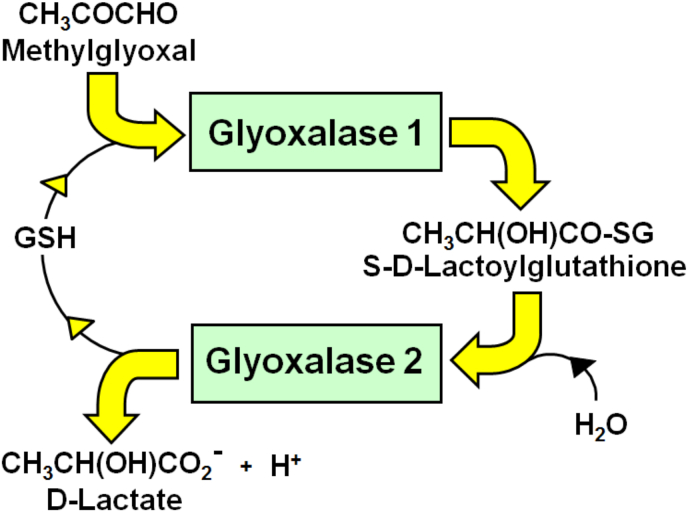
**The glyoxalase system.** The glyoxalase system is in the cytosol of all cells and catalyses the GSH-dependent conversion of methylglyoxal to d-lactate, via intermediate, *S*-d-lactoylglutathione [26].

Increased formation of MG-derived AGEs has been linked to the aging and aging-related disorders – reviewed in [[25], [26], [27], [28]]. MG-derived AGE content of human lens and skin protein increases with age [[29], [30], [31], [32]]. MG modifies proteins to form MG-H1 and lysine-derived N_ε_(1-carboxyethyl)lysine (CEL) – including in skeletal muscle [33]. MG is metabolized mainly by glyoxalase 1 (Glo1) of the cytosolic glyoxalase system [26] (Fig. 1). Protein glycation by MG increases with aging likely as a consequence of increased MG formation in age-related insulin resistance and dysglycemia, and decreased MG metabolism by decline of Glo1 activity in aging [[34], [35], [36]]. In the nematode *Caenorhabditis elegans*, overexpression of Glo1 increased median and maximum lifespan by *ca.* 30% and silencing of Glo1 decreased lifespan by *ca.* 50–60% [37]. In rats, caloric restriction was associated with improved metabolic health, longevity and decreased CEL content of heart mitochondrial protein [38]. Finally, treatment of overweight and obese subjects with a dietary supplement inducing increased expression of Glo1 and decreased MG and MG-related AGE formation, producing improved metabolic health - including correction of insulin resistance and improvement of dysglycemia [34].

In this study, we analysed protein glycation, oxidation and nitration adducts in skeletal muscle, plasma and urine of young and aged HSA-mUCP1 mice and WT controls - 12 and 70 weeks old, respectively. The outcome revealed a decrease in AGEs formed by MG, suggesting an association of the healthy aging response with decreased MG protein glycation.

## 2 Materials and methods

### 2.1 Wild type and transgenic mice

HSA-mUCP1 mice were generated as described previously [3]. Experiments were performed with male HSA-mUCP1 and WT controls maintained on a mixed C57BL/6-CBA background. All animal experiments were performed in compliance with the German animal protection law (TierSchG). The mice were housed and handled in accordance with good animal practice as defined by FELASA (www.felasa.eu/guidelines.php) and the national animal welfare body GV-SOLAS (www.gv-solas.de/index.html). The animal welfare committees of the DIfE as well as the local authorities (Landesamt für Umwelt, Gesundheit und Verbraucherschutz, Brandenburg) approved all animal experiments. Mice were housed in groups with *ad libitum* access to a standard chow diet and water. At 12 and 70 weeks of age, mice were euthanized in the morning 2 h after food withdrawal. Skeletal muscle tissue samples (quadriceps, 20–25 mg), plasma (100 μl) and urine (from 70 week-old only) wild type and HSA-mUCP1 mice were collected and stored at – 80 °C until analysis, with shipment to the collaborating UK-based laboratory in dry ice. Quadriceps was chosen as representative of predominantly fast/glycolytic muscles which are most affected by mitochondrial uncoupling and constitute the bulk of murine skeletal muscle [11].

### 2.2 Protein damage marker assessment

Glycation, oxidation and nitration adduct content of skeletal muscle and plasma protein was quantified in exhaustive enzymatic digests by stable isotopic dilution analysis LC-MS/MS, with correction for autohydrolysis of hydrolytic enzymes as described [39]. Related free adducts (glycated, oxidized and nitrated amino acids) were determined in the ultrafiltrates of urine samples (100 μl) by microspin ultrafiltration (3 kDa cut-off) at 4 °C. Skeletal muscle tissue samples (*ca.* 5 mg wet weight) were homogenized in 10 mM sodium phosphate buffer, pH 7.4 and 4 °C, and membranes and fibrils sedimented by centrifugation (20,000 g, 30 min, 4 °C). The supernatant was diluted to 500 μl with water and washed by ultradiafiltration over a 10 kDa ultrafilter: 4 cycles of concentration to 50 μl and dilution to 500 μl with water over the microspin ultrafilter at 4 °C. The final washed protein was delipidated and hydrolysed enzymatically as described [39,40]. Plasma protein was prepared similarly from 100 μl plasma. Total protein in the final concentrate was determined by Bradford assay and an aliquot of protein (100 μg) was digested by exhaustive enzymatic hydrolysis under aseptic, antioxidant conditions using a PAL sample autoprocessor (CTC Analytics, Zwingen, Switzerland). Protein hydrolysate (25 μl, 32 μg equivalent) or ultrafiltrate was mixed with stable isotopic standard analytes and analysed by LC-MS/MS using an Acquity™ UPLC system with a Quattro Premier tandem mass spectrometer (Waters, Manchester, U.K.) [21,39]. Samples were maintained at 4 °C in the autosampler during batch analysis. The columns were: 2.1 × 50 mm and 2.1 mm × 250 mm, 5 μm particle size Hypercarb™ (Thermo Scientific), in series with programmed switching, at 30 °C. Chromatographic retention is necessary to resolve oxidized analytes from their amino acid precursors to avoid interference from partial oxidation of the latter in the electrospray ionization source of the mass spectrometric detector. Analytes were detected by electrospray positive ionization and mass spectrometry multiple reaction monitoring (MRM) mode where analyte detection response is specific for mass/charge ratio of the analyte molecular ion and major fragment ion generated by collision-induced dissociation in the mass spectrometer collision cell. The ionization source and desolvation gas temperatures were 120 °C and 350 °C, respectively, cone gas and desolvation gas flow rates were 99 and 900 l/h and the capillary voltage was 0.60 kV. Argon gas (5.0 × 10^−3^ mbar) was in the collision cell. For MRM detection, molecular ion and fragment ion masses and collision energies optimized to ±0.1 Da and ±1 eV, respectively, were programmed [21,39]. Analytes determined were: glycation adducts – FL, CML, CEL, hydroimidazolones derived from glyoxal, MG and 3-DG, G-H1, MG-H1 and 3DG-H, respectively, glyoxal-derived N_ω_-carboxymethylarginine (CMA), PENT, AASA, GSA and 3-NT. Chemical structures and physiological significance of these analytes have been described elsewhere [20,21]. Amino acids quantified were: arg, lys, tyr, and val (valine is determined in protein hydrolysates for the protease autohydrolysis correction) [39]. Analyte adduct residues were normalized to their amino acid residue precursors and given as mmol/mol amino acid modified; and related free adducts in urine are given in nmol/mg creatinine. Creatine was determined in urine by LC-MS/MS as described [41].

### 2.3 High mass resolution proteomics analysis of skeletal muscle

Cytosolic protein extracts of skeletal muscle from 70-week old Tg and WT control mice were analysed by high resolution Orbitrap mass spectrometry of tryptic digests [42]. Cytosolic protein extracts were prepared and washed by ultradiafiltration over 10 kDa ultrafilters, as described above. A similar sample of [^13^C_6_]lysine-labelled mouse skeletal muscle was processed similarly to produce [^13^C_6_]lysine-labelled peptide digest for internal standardization. An aliquot of cytosolic protein extract (89 μg, 50 μl) was treated with dithiothreitol (6 μl, 6 mM) and incubated at 37 °C in the dark for 30 min; and then treated with iodoacetamide (5.9 μl, 10.8 mM) and incubated at 37 °C in the dark for 30 min. Residual iodoacetamide was then quenched by further addition of dithiothreitol (5.9 μl 6 mM) and incubated at 37 °C in the dark for 30 min. An aliquot of Lys-C protease (1 mg/ml, 5 μl) in 500 mM ammonium bicarbonate, pH 8.0, was added and incubated for 1 h at 37 °C. Then *p*-tosyl-phenylalanyl chloromethyl ketone (TPCK)-treated trypsin (1 mg/ml, 5 μl) in 1 mM calcium chloride/500 mM ammonium bicarbonate, pH 8.0, was added and samples were incubated at 37 °C for 5 h in the dark. Finally, the reaction was stopped by adding 10% trifluoroacetic acid (5 μl) in water. The sample was lyophilized to dryness and re-suspended in an aliquot (100 μl) 0.1% formic acid in water.

The tryptic digest samples were submitted to the Mass Spectrometry and Proteomics Facility, University of Warwick, for label-free proteomic quantitation analysis. Reversed phase nanoflow liquid chromatography-mass spectrometry for global protein identification was performed on an Orbitrap mass spectrometer (Fusion™ Tribrid™, ThermoFisher Scientific) equipped with a microspray source operating in positive ion mode. The column used was: an Acclaim PepMap μ-pre-column cartridge (trap), 300 μm i.d. x 5 mm, 5 μm particle size, 100 Å pore size, fitted to an Acclaim PepMap RSLC 75 μm i.d. x 50 cm, 2 μm particle size, 100 Å pore size main column (ThermoFisher Scientific). It was installed on an Ultimate 3000 RSLCnano system (Dionex). An aliquot of sample (5 μl) containing a 1: 1 mixture of nature isotopic abundance and [^13^C_6_]lysine-labelled digests (*ca.* 1 μg equivalent) was injected. After injection, the peptides were eluted off of the trap onto the analytical column. Mobile phases were: A - 0.1% formic acid in water, and B - 0.1% formic acid in acetonitrile. The flow rate was programmed at 0.3% to 35% 220 min. Mobile phase B was then increased from 35% to 80% in 5 min before being brought back quickly to 3% in 1 min. The column was equilibrated at 3% of mobile phase B for 15 min before the next sample. Peptides were eluted directly (300 nl min^−1^) via a Triversa Nanomate nanospray source (Advion Biosciences, NY) into the Orbitrap mass spectrometer. Survey scans of peptide precursors from 350 to 1500 *m/z* were performed at 120 K resolution (at 200 *m/z)* with automatic gain control (AGC) 4 × 10^5^. Precursor ions with charge state 2–7 were isolated in 1.6 Th intervals in the quadrupole and subjected to high energy collision dissociation fragmentation programmed to 35% and fragments ions detected by rapid scan MS analysis in the ion trap; the AGC was set to 1 × 10^4^ and the max injection time was 200 ms. Dynamic exclusion duration was set to 45 s with a 10 ppm tolerance around the selected precursor and its isotopes. Monoisotopic precursor selection was turned on. The instrument was run in top speed mode with 2 s cycles. Sequence information from the MS/MS data was managed using MSConvert in ProteoWizard Toolkit (version 3.0.5759) [43] and searched with Mascot engine (Matrix Science, version 2.5.0) against *Mus musculus* protein sequence database (http://www.uniprot.org/) assuming enzyme tryptic digestion to determine levels of false-positive peptide identifications; spectra were also searched against the corresponding reverse database, the common Repository of Adventitious Proteins Database (http://www.thegpm.org/cRAP/index.html). Search parameters for Precursor mass and product ions tolerance were, respectively, ±5 ppm and ±0.8 Da, with allowance made for two missed trypsin cleavages, fixed modification of cysteine through carbamidomethylation and methionine oxidation. Only fully tryptic peptide matches were allowed. Scaffold (version Scaffold 4.3.2, Proteome Software Inc.) was used to validate MS/MS based peptide and protein identifications from MS/MS sequencing results. Peptide identifications were accepted if they could be established at greater than 95.0% probability by the Scaffold Local FDR algorithm and contained at least 2 identified unique peptides; probabilities assigned by the Protein Prophet algorithm [44]. Peptide abundances were normalized to [^13^C_6_]lys-labelled internal standards; mean SD for detection of labelled standards was 14.9%, WT 19.9% and HSA-mUCP1 27.2%. For [^13^C_6_]lys-labelled standard protein abundances, correlation coefficient r values between runs for peptide abundance were in the range 0.990–0.999 (P < 0.001).

### 2.4 Other measurements

The concentrations of MG, reduced glutathione (GSH) and oxidized glutathione (GSSG) in mouse skeletal muscle were assayed by LC-MS/MS and the activity of glyoxalase 1 (Glo1) was assayed spectrophotometrically, as described [34,45,46]. Protein thiols were deduced by determining total cellular thiols by derivatisation with 5,5′-dithiobis(2-nitrobenzoic acid) (DTNB) [47] and subtracting cellular GSH content [48]. Briefly, muscle tissue (*ca*. 1 mg wet weight) was homogenized on ice in 10 mM sodium phosphate buffer, pH 7.0, cell membranes sedimented by centrifugation (20,000 g, 30 min, 4 °C). DTNB solution (1 mM in 100 mM sodium phosphate buffer, pH 7.4, with 0.2 mM diethylenetriaminepenta-acetic acid; 125 μl) was added to water (100 μl) and tissue extract (25 μl). The increase in absorbance at 405 nm after 20 min was used to deduce thiol content, calibrated by assay of 0–16 nmol GSH. Protein thiol content is given as nmol per mg tissue wet weight.

### 2.5 Materials

L-Lactic dehydrogenase from bovine heart, type III (Cat# L2625), 1,2-diaminobenzene, sublimed (Cat# 694975), pepsin from porcine stomach mucosa (Cat# P6887), prolidase from porcine kidney (Cat# P6675), pronase E − type XIV from *Streptomyces griseus* (Cat# P5147), leucine aminopeptidase - type VI from porcine kidney (Cat# L6007), DL-dithiothreitol (Cat# 43819), iodoacetamide (Cat# I1149), N-*p*-tosyl-l-phenylalanine chloromethyl ketone-treated trypsin (Cat# 4352157-1 KT) and endoproteinase Lys-C - sequencing grade from *Lysobacter enzymogenes* (Cat# 11047825001) were from Merck (Poole, Dorset, U.K.). Radio-immunoprecipitation assay (RIPA) buffer (Cat# 9806) was from Cell Signaling Technology (Leiden, The Netherlands). Glycated, oxidized and nitrated amino acids, natural isotopic abundance and stable isotopic standards, were purchased from Cambridge Isotope Laboratories, Inc, Tewksbury, MA, USA – where available, or otherwise prepared in-house, as previously described [18,33,49]. [^13^C_6_]Lysine-labelled mouse skeletal muscle (>97 atom %, Product no. 252923908) was from Silantes, Munich, Germany.

### 2.6 Statistical analysis

Data are mean ± SD for parametric data and median (upper – lower quartile) for non-parametric data, unless otherwise stated. Significance testing was by Student's t-test and Mann-Whitney *U* test (for 2 two groups), by one-way ANOVA and Kruskal-Wallis test (for 4 groups) for parametric and non-parametric data, respectively, and correlation analysis by the Pearson method. P < 0.05 was considered statistically signiﬁcant. Statistical analyses were performed using SPSS (version 24.0, Armonk, NY, USA).

## 3 Results

### 3.1 Physiology of young and aged HSA-mUCP1 and WT control mice

Both young and aged HSA-mUCP1 mice had over 30% reduced body weight compared to WT controls. This is due to a decreased body size concomitant with decreased lean and fat mass as shown previously [9,50,51]. Nose to anus body length of adult HSA-mUCP1 mice was reduced by about 0.5 cm (5%) compared to WT [9,50]. Skeletal muscle (quadriceps) gene expression of UCP1 was increased *ca.* 45-fold in adult HSA-mUCP1 mice, compared to WT controls, and UCP1 protein expression - not detectable in WT - was *ca.* 14-fold lower than in brown adipose tissue [9]. Of note, HSA-mUCP1 mice displayed a pronounced muscle atrophy. Absolute and relative muscle (quadriceps) weight of aged mice was significantly reduced compared to WT (Table 1), in line with the previously reported uncoupling induced decrease of grip strength, muscle mass, and muscle fibre size (diameter) of these mice [52,53].

**Table 1:** Mouse morphometrics.

Genotype	Young		Aged		Significance P
	Wild-type	HSA-UCP1	Wild-type	HSA-UCP1	
Body weight (g)	28.5 ± 0.3	19.5 ± 0.3	37.6 ± 1.8	23.3 ± 0.7	<0.0001
Quadriceps (g)	ND	ND	0.403 ± 0.030	0.167 ± 0.001	<0.0001
Quadriceps (% BW)	ND	ND	0.55 ± 0.02	0.37 ± 0.01	<0.0001

Data are mean ± SEM (n = 7–8). Significance: *Students t-test,* HSA-mUCP1 vs WT control of same age. Abbreviations: BW, body weight; and ND, not determined.

### 3.2 Protein glycation, oxidation and nitration adducts in skeletal muscle, plasma protein and urine of HSA-mUCP1 and WT control mice

**Fig. 2:**
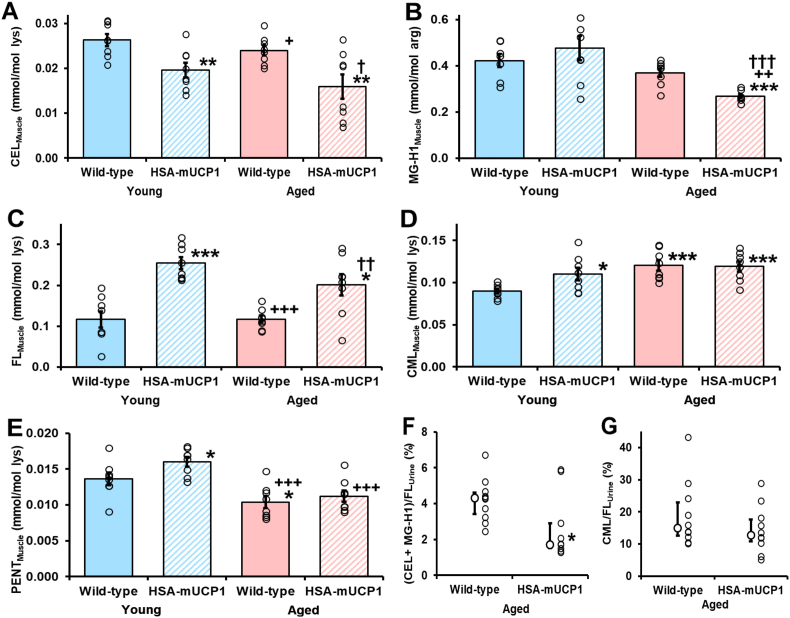
**Changes in protein glycation status in young and aged HSA-mUCP1 transgenic mice, with respect to wild-type controls.** Glycation adduct residue content of skeletal muscle protein: A. CEL, B. MG-H1, C. FL, D. CML and E. PENT. Urinary excretion of glycation free adducts as a percentage of FL free adduct flux: F. CEL + MG-H1 and G. CML. Key: filled bars, WT; hatched bars, HSA-mUCP1; pastel blue bars, young; and pastel red bars, aged. Data are mean ± SEM (n = 8 for A – E; n = 10 for F and G). Significance: *, ** and ***, P < 0.05, P < 0.01 and P < 0.001 with respect to young WT control; +, + + and + + +, P < 0.05, P < 0.01 and P < 0.001 with respect to young HSA-mUCP1 mice control; and †, †† and †††, P < 0.05, P < 0.01 and P < 0.001 with respect to aged WT control; *Student's t-test*. For ANOVA analysis, see Supplementary Table S1. Two outliers were removed from B. (Young WT, 0.739 and aged HSA-mUCP1, 0.588). (For interpretation of the references to colour in this figure legend, the reader is referred to the Web version of this article.)

For skeletal muscle, the CEL contents of young and aged HSA-mUCP1 mice were decreased, 25% and 40%, respectively, with respect to young WT controls. The MG-H1 content of aged HSA-mUCP1 mice was decreased 36%, with respect to young WT control. The CEL and MG-H1 content of aged HSA-mUCP1 mice were decreased 34% and 27%, with respect to aged WT control (Fig. 2A and B). In contrast, FL contents of young and aged mice HSA-mUCP1 were increased 117% and 72%, respectively, compared to WT controls (Fig. 2C). There were also minor increases in CML content of young HSA-mUCP1, aged WT and aged HSA-mUCP1 mice, compared to young WT controls, +23%–34%; and no change between aged HSA-mUCP1 and WT controls (Fig. 2D). The PENT content was increased in young HSA-mUCP1 mice, compared to young WT mice, and decreased in aged WT mice; and no change of PENT in aged HSA-mUCP1 compared to aged WT mice (Fig. 2E). Hydroimidazolone 3DG-H was increased 36–40% in aged WT mice and young and aged HSA-mUCP1 mice, with respect to young WT mice, and was also unchanged in aged HSA-mUCP1 mice, with respect aged WT mice. CMA was increased 31% in skeletal muscle of young HSA-mUCP1 mice, with respect to WT controls, and was unchanged in aged WT and HSA-mUCP1 mice. AASA was unchanged in young and aged HSA-mUCP1 mice and aged WT mice, compared to young WT mice; and GSA was below the limit of detection, <0.012 mmol/mol lys, in all study groups. 3-NT was decreased 19% in aged HSA-mUCP1, with respect to aged WT controls (Supplementary Table S1).

We also investigated changes in the glycation, oxidation and nitration adduct residue content in plasma protein. Overall, there were few changes in plasma protein modifications in HSA-mUCP1 and aged mice. Where changes were found – such as decreased FL content in aged WT and HSA-mUCP1 mice, they did not correlate with concomitant changes in skeletal muscle (Supplementary Table S2).

Changes in whole body flux of protein glycation were explored by measurement of flux of glycated amino acids in urine of aged WT and HSA-mUCP1 mice (Supplementary Table S3). There were no changes in urinary flux of analytes in HSA-mUCP1 mice compared to WT controls. Normalized to the flux of FL free adduct as a marker of glucose exposure, there was a 60% decrease in urinary MG-derived AGEs – (CEL + MG-H1)/FL ratio. Urinary CML/FL ratio was unchanged (Fig. 2F and G).

**Fig. 3:**
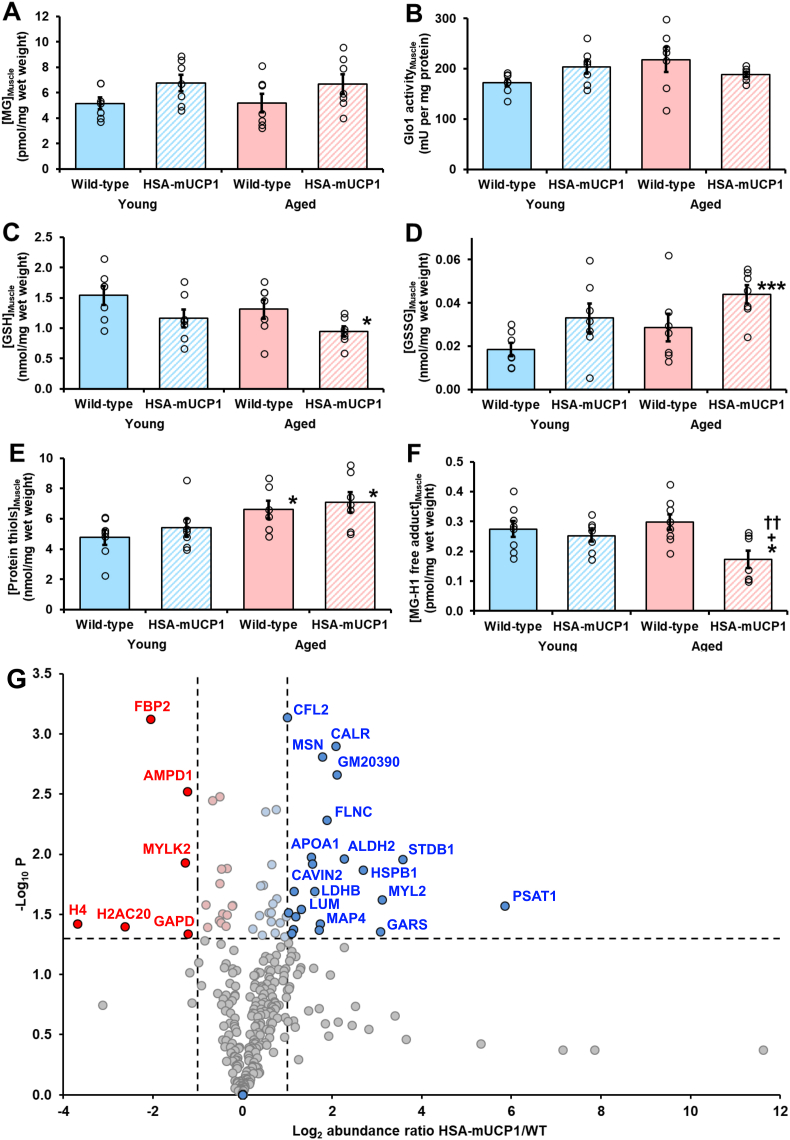
Studies on the mechanism of decrease of methylglyoxal-modified protein in skeletal muscle aged HSA-mUCP1 mice. Skeletal muscle metabolites and Glo1 activity: A. Concentration of MG; B. Activity of Glo1; C. Concentration of GSH; D. Concentration of GSSG; E. Concentration of protein thiols; and F. Concentration of MG-H1 free adduct. Data are mean ± SEM (n = 7). Key: filled bars, WT; hatched bars, HSA-mUCP1; pastel blue bars, young; and pastel red bars, aged. Significance: * and ***, P < 0.05 and P < 0.001 with respect to young WT control; +, P < 0.05 with respect to young HSA-mUCP1 mice control; and ††, P < 0.01 with respect to aged WT control; *Student's t-test*. For 4 study group comparison, P < 0.05 for GSH, GSSG, protein thiols and MG-H1 free adduct; *ANOVA*. Outliers removed: Protein thiols - aged WT, 15.6; and MG-H1 free adduct - young HSA-mUCP1, 0.511. G. Skeletal muscle proteomics. A volcano plot of change in abundance in cytosolic extracts of skeletal muscle of aged HSA-mUCP1 mice. Each circle represents a protein identified. The horizontal line represents the P = 0.05 significant threshold (-log_10_ = 1.301). The vertical lines indicate 2-fold decrease and increase in protein abundances. Proteins of decreased and increased abundance shown in red and blue filled circle, respectively. Total number of proteins shown: 302. Abbreviated names of proteins are given alongside data points where possible without loss of clarity. (For interpretation of the references to colour in this figure legend, the reader is referred to the Web version of this article.)

We explored the mechanism of decreased MG-derived AGEs in skeletal muscle protein of aged HSA-mUCP1 mice, compared to WT controls. MG-related variables were measured in skeletal muscle of young and aged WT and HSA-mUCP1 mice. The concentration of MG in skeletal muscle of young WT mice was 5.16 ± 1.22 pmol per mg tissue wet weight and was unchanged in aged WT mice and young and aged HSA-mUCP1 mice (Fig. 3A). Glo1 activity of skeletal muscle was 172 ± 20 mU per mg protein in young WT mice and also was unchanged in aged WT mice and young and aged HSA-mUCP1 mice. The *in situ* activity of Glo1 is dependent in the concentration of GSH and MG is also bound reversibly by protein thiols [26], so we also determined the concentrations of GSH, GSSG and protein thiols in skeletal muscle of WT and HSA-mUCP1 mice. The concentration of GSH in skeletal muscle of young WT mice was 1.54 ± 0.41 nmol per mg wet weight. It was unchanged in young HSA-mUCP1 mice and aged WT mice but was decreased 39% in aged HSA-mUCP1 mice, with respect to young WT control but not with respect to aged WT control (Fig. 3C). The concentration of GSSG in skeletal muscle of young WT mice was 0.018 ± 0.009 nmol per mg wet weight. It was unchanged in young HSA-mUCP1 mice and aged WT mice but was increased *ca.* 2-fold in aged HSA-mUCP1 mice, with respect to young WT controls but not with respect to aged WT controls (Fig. 3D). Skeletal muscle concentration of GSSG remained very low level as a percentage of total GSH (<1.5%), indicating skeletal muscle cytoplasm remained strongly reduced in all study groups. The concentration of protein thiols in skeletal muscle of was 4.77 ± 1.35 nmol per mg tissue wet weight – *ca.* 3-fold higher than the concentration of GSH, and was unchanged in young HSA-mUCP1 mice. It was increased 13% and 31% in aged WT and aged HSA-mUCP1 mice, respectively, compared to young WT mice; and was unchanged in aged HSA-mUCP1 mice, with respect to aged WT mice (Fig. 3E). This indicated there was no change in exposure to MG and concentrations of GSH, GSSG and protein thiols of skeletal muscle of aged HSA-mUCP1 mice, compared to aged WT controls. A likely explanation of decreased MG-modified protein in skeletal muscle was, therefore, increased proteolysis of MG-modified proteins. To gain evidence of this, we measured the concentration of MG-H1 free adduct in cytosolic extracts of skeletal muscle. The concentration of MG-H1 free adduct in skeletal muscle of young WT mice was 0.275 ± 0.076 pmol per mg tissue wet weigh (*ca.* 260 nM). It was unchanged in young HSA-mUCP1 mice and aged WT mice but was decreased 42% in aged HSA-mUCP1 mice, with respect aged WT mice (Fig. 3F).

### 3.3 Proteomics analysis of skeletal muscle of aged HSA-mUCP1 and WT control mice

We performed proteomics analysis of cytosolic protein extracts of skeletal muscle to identify changes of protein abundances in HSA-mUCP1 mice associated with healthy aging in aged HSA-mUCP1 mice and locations of the major MG-derived AGE, MG-H1, in aged WT mice.

We identified and quantified 302 proteins common to the skeletal muscle proteome of aged WT and HSA-mUCP1 mice. MG-H1 modification was detected on 6 proteins (sequence location): myosin-binding protein C (R432), myosin regulatory light chain 11 (MYL11) (R130), creatine kinase B (R172), protein kinase C/casein kinase II substrate protein 3 (PACSIN3) (R137), β-actin (R210) and γ-actin (R290). Two of these, MYL11 and PACSIN3, were also detected in aged HSA-mUCP1 mice. Thirty-nine proteins had increased abundance in aged HSA-mUCP1 mice, compared to WT controls, with abundance increases from 17% to 58-fold (Table 2); and there were 20 proteins of decreased abundance, with decreases from 15 to 92% (Table 3). Protein abundance changes are presented in a volcano plot (Fig. 3G). Major abundance increases were linked to increased serine synthesis (PSAT1), glycogen storage (STBD1) and muscle contraction (MLRV). The was also a 6-fold increase in heat shock protein beta-1 (HSPB1/HSP25) – the major chaperone protein of skeletal muscle [54]. Other chaperone proteins were also increased: heat shock 70 kDa protein 4 (HSPA4; +97%), stress-70 protein, mitochondrial (HSPA9; +65%) and T-complex protein 1 subunit zeta (CCT6A; +54%) – a component of the chaperonin-containing T-complex (TRiC). Caveolae-associated proteins 1 and 2 were also increased (2 and 3-fold).

**Table 2:** Proteins of increased abundance change in cytosolic extracts of skeletal muscle of aged HSA-mUCP1 mice, with respect to aged wildtype controls.

No	Accession no	Description	Abundance ratio (HSA-mUCP1/WT)	P-value	Pathway involvement/function
1.	Q3U6K9	Phosphoserine aminotransferase (PSAT1)	57.57	0.027	Serine biosynthesis
2.	Q8C7E7	Starch-binding domain-containing protein 1 (STBD1)	11.91	0.011	Glycogen storage
3.	P51667	Myosin regulatory light chain 2 (MLRV)	8.65	0.024	Muscle contraction
4.	Q9CZD3	Glycine--tRNA ligase	8.39	0.044	Protein translation
5.	P14602	Heat shock protein beta-1 (HSPB1)	6.44	0.014	Major skeletal muscle chaperone
6.	P47738	Aldehyde dehydrogenase, mitochondrial	4.79	0.011	Metabolizes lipid peroxidation products
7.	E9PZF0	Nucleoside diphosphate kinase	4.28	0.002	
8.	P14211	Calreticulin	4.22	0.001	Calcium-binding chaperone
9.	Q8VHX6	Filamin-C	3.69	0.005	Sarcomere assembly and organization
10.	P26041	Moesin	3.43	0.002	Connects the actin cytoskeleton to the plasma membrane
11.	P27546	Microtubule-associated protein 4	3.31	0.038	Microtubule assembly
12.	P60843	Eukaryotic initiation factor 4A-I	3.28	0.042	Protein translation
13.	P16125	l-Lactate dehydrogenase B	3.03	0.020	Glycolysis
14.	Q63918	Caveolae-associated protein 2	2.92	0.012	Caveolar biogenesis and morphology
15.	Q00623	Apolipoprotein A-I	2.88	0.011	Lipid and cholesterol transport in HDL
16.	P51885	Lumican	2.46	0.029	Caveolae formation and organization
17.	G3UXL2	Ribose-phosphate diphosphokinase	2.27	0.033	
18.	P99027	60S acidic ribosomal protein P2 (Eukaryotic initiation factor 4A-I)	2.22	0.020	Peptide elongation in protein synthesis
19.	P04117	Fatty acid-binding protein, adipocyte	2.18	0.042	Lipid transport protein
20.	O54724	Caveolae-associated protein 1	2.12	0.045	Caveolae formation and organization
21.	P09542	Myosin light chain 3	2.03	0.031	Muscle contraction
22.	P45591	Cofilin-2	2.00	0.001	Controls reversible actin polymerization
23.	Q61316	Heat shock 70 kDa protein 4 (HSPA4)	1.97	0.033	Chaperone protein
24.	Q91X72	Hemopexin	1.91	0.048	Heme transport
25.	G3UY93	Valine--tRNA ligase	1.78	0.037	Protein translation
26.	Q99NF7	Protein-serine/threonine phosphatase	1.70	0.023	
27.	K3W4S6	Glycogenin-1	1.68	0.004	Glycogen synthesis
28.	P38647	Stress-70 protein, mitochondrial (HSPA9)	1.65	0.045	Chaperone protein
29.	Q08642	Protein-arginine deiminase type-2	1.59	0.026	
30.	P97457	Myosin regulatory light chain 2	1.56	0.012	Muscle contraction
31.	Q6PF96	Electron transfer flavoprotein-ubiquinone oxidoreductase, mitochondrial	1.55	0.036	
32.	P80317	T-complex protein 1 subunit zeta	1.54	0.031	Component of the chaperonin-containing T-complex (TRiC)
33.	Q9JLV1	BAG family molecular chaperone regulator 3	1.49	0.031	Co-chaperone for HSP70 and HSC70 chaperone proteins
34.	Q9CQ65	*S*-methyl-5′-thioadenosine phosphorylase	1.42	0.004	
35.	P54822	Adenylosuccinate lyase	1.38	0.018	
36.	P20801	Troponin C	1.35	0.047	Muscle contraction
37.	P05977	Myosin light chain 1/3	1.32	0.018	Formation and/or maintenance of myofibers
38.	P56480	ATP synthase subunit beta, mitochondrial	1.29	0.032	Cell respiration
39.	Q91ZJ5	UTP--glucose-1-phosphate uridyltransferase	1.17	0.041	Glycogen synthesis

**Table 3:** Proteins of highest decreased abundance change in cytosolic extracts of skeletal muscle of aged HSA-mUCP1 mice, with respect to aged wildtype controls.

No	Accession no	Description	Abundance ratio (HSA-mUCP1/WT)	P-value	Pathway involvement/function
1.	P62806	Histone H4	0.08	0.038	Nucleosome
2.	Q64523	Histone H2A type 2-C	0.16	0.040	Nucleosome
3.	P70695	Fructose-1,6-bisphosphatase isozyme 2	0.24	0.001	Regulation of glycolysis
4.	Q8VCR8	Myosin light chain kinase 2	0.41	0.012	Muscle contraction
5.	Q3V1D3	AMP deaminase 1	0.42	0.003	
6.	P16858	Glyceraldehyde-3-phosphate dehydrogenase	0.43	0.046	Glycolysis
7.	Q9CZ30	Obg-like ATPase 1	0.57	0.026	ATP hydrolysis
8.	P45376	Aldose reductase	0.58	0.040	
9.	P12787	Cytochrome *c* oxidase subunit 5A, mitochondrial	0.62	0.004	Cell respiration
10.	Q9ET78	Junctophilin-2	0.70	0.035	
11.	E9Q1W3	Nebulin	0.70	0.003	Component of the skeletal muscle thin filament
12.	Q9WUB3	Glycogen phosphorylase	0.70	0.017	Glycogenolysis
13.	E9Q8P5	PDZ and LIM domain protein 5	0.71	0.037	
14.	Q564E2	l-lactate dehydrogenase-A	0.72	0.013	Glycolysis
15.	P50247	Adenosylhomocysteinase	0.73	0.031	
16.	Q9DCZ1	GMP reductase 1	0.77	0.039	
17.	P10649	Glutathione *S*-transferase M1	0.78	0.032	Metabolizes lipid peroxidation products
18.	P47754	F-actin-capping protein subunit alpha-2	0.78	0.013	
19.	P58771	Tropomyosin alpha-1 chain	0.84	0.027	Regulation of muscle contraction
20.	P16015	Carbonic anhydrase 3	0.85	0.027	

## 4 Discussion

In this study we found the HSA-mUCP1 mouse model of healthy aging was associated with decreased MG-derived AGE residues and increased FL residues and in skeletal muscle proteins in young and aged mice. Previous studies of fructosamine 3-phosphokinase deficient mice indicated there is no aging phenotype associated with increased FL glycation [55]. Therefore, healthy aging in the HSA-mUCP1 mouse may be mediated, at least in part, by decreased MG-derived AGE accumulation.

The decrease of MG-derived AGEs, CEL and MG-H1, in skeletal muscle aging of HSA-mUCP1 was not linked to decrease in MG concentration or increase of GSH and protein thiol concentrations or activity of Glo1. The effect may rather be due increased proteolysis and turnover of MG-modified proteins. There was a 3-fold increase of HSPB1 and increases in other chaperone proteins in skeletal muscle of aged HSA-mUCP1 mice and this likely increases myocyte proteolysis, decreasing MG-H1 content of skeletal muscle protein. The increased abundance of HSPB1 in skeletal muscle of HSA-mUCP1 mice was previously validated by immunoblotting [50]. HSPB1 is highly expressed in skeletal muscle, is induced by metabolic stress and increased contractile activity [56] and deficiency induced myofibrillar structure abnormalities [57]. Our previous studies [9,11,52] showed that increased mitochondrial uncoupling by UCP1 in HSA-mUCP1 mice was associated with activation of AMPK and increased phosphorylation of the eukaryotic initiation factor-2α (eIF2α) of the integrated stress response [58]. Increased phosphorylated eIF2α increased expression of sensor, inositol requiring enzyme-1α (IRE1α), and transcription factors ATF4, CHOP and ATF6 of the endoplasmic reticulum unfolded protein response (UPR). It also increased expression of ATF4, ATF5 and CHOP of the mitochondrial UPR [59]. This increases proteostasis surveillance, with downstream chaperone-targeted ubiquitin ligase protein ubiquitination for proteasomal degradation. Our recent studies indicate MG-modified proteins are often misfolded due to a positively-charged arginine residue being replaced with a hydrophobic uncharged MG-H1 residue, and activate the UPR [23,48,60]. Ubiquitin ligase HUWE1 was implicated in the ubiquitination and degradation of MG-modified proteins [48]. HSPB1 has expression positively regulated by ATF5 [61] and binds HUWE1 to direct it to ubiquitination substrates [62]. Accordingly, the signaling involved in decreased MG-modified protein in skeletal muscle of HSA-mUCP1 mice is likely increased HSPB1-directed HUWE1 ubiquitination through eIF2α-mediated, ATF5-induced increased expression of HSPB1.

Beneficial effects of increased skeletal muscle proteolysis are muscle restructuring and recovery of functional deficits from MG-modified proteins. For the MG-modified proteins identified, myosin-binding protein C is a thick filament protein involved in the regulation of muscle contraction, binding to myosin through two immunoglobulin-like domains. The MG modification site R432 lies in the 95 residue linker between these domains of uncertain function [63]. MYL11 is involved in regulation of muscle contraction [64]. Activity of creatinine kinase-B declines in aging and is inhibited by MG modification [65,66]. PACSIN3 is involved in caveolae and muscle repair [67], and β-actin and γ-actin are modified by MG in the Mg^2+−^ADP binding site and ubiquitination site, respectively, which likely impairs functional activity and degradation, respectively [68]. Decrease of this and proteomic restructuring of skeletal muscle may provide for improved metabolic and muscular health in aging. Caveolae-associated proteins 1 and 2 were also increased and are involved in mechanoprotection and repair of muscle fibers [69,70].

Whole body exposure to MG-derived AGEs, assessed by urinary excretion of free adducts normalized to FL free adduct, was decreased 60% in aged HSA-mUCP1 mice whereas CML/FL ratio (a marker of oxidative stress [71]) was unchanged. This indicates that for a given glycemic exposure, aged HSA-mUCP1 mice produce markedly lower MG-derived AGEs than WT controls. In mouse muscle, the period of lowest and highest glycolytic intermediates occurs after periods of physical activity and sedentary behaviour, respectively [72]. Decreased fasting plasma glucose in aged HSA-mUCP1 mice may produce lower glucose disposal in the fasting phase and decreased unscheduled glycolysis and formation of MG [73]. This may lead to the decreased total body MG exposure and formation of MG-derived AGEs.

Increased FL content of skeletal muscle protein of young and aged HSA-mUCP1 mice is indicative of increased cellular concentration of glucose in skeletal muscle of HSA-mUCP1 mice; *cf*. increase of FL residue content of cytoplasmic proteins of endothelial cells incubated in high glucose concentration [60]. This is consistent with previous reports of increased glucose uptake and GLUT4 protein activity in skeletal muscle of HSA-mUCP1 mice [9,10]. There was expected related increases of FL-derived AGEs, CML and 3DG-H [14,74], and increase of CML in skeletal muscle of aged WT mice, consistent with increased oxidative stress. The increase of PENT in young HSA-mUCP1 mice may indicate increased PPP activity - for which evidence was found previously [11] and PENT was decreased in aged WT mice – likely reflecting the decline of PPP activity in skeletal muscle in old age [75]. Decreased FL, 3DG-H and AASA residue content of plasma protein in aged WT mice may relate to increased capillary permeability and albumin transcapillary escape rate in aged mice where plasma protein has increased dwell time in the lower glycating and oxidizing environment of interstitial fluid [76].

## 5 Conclusions

Aged HSA-mUCP1 mice had decreased MG-derived AGEs in skeletal muscle and decreased whole body MG-derived AGE exposure which is likely linked to the healthy aging phenotype. Decreased MG-derived AGEs in skeletal muscle is likely produced by increased proteolysis and proteostasis surveillance of the skeletal muscle proteome. In previous studies, WT and HSA-mUCP1 mice exhibited a similar level of physical activity but HSA-mUCP1 mice had smaller muscle mass [3,7,11]. This greater physical activity per unit of muscle mass may provide for increased and efficient muscle activity, maintaining high insulin sensitivity, decreased fasting plasma glucose with decreased propensity to form MG [73]. A similar decreased exposure to MG-derived AGEs may be achieved clinically with dietary supplement inducer of Glo1 expression, *trans*-resveratrol and hesperetin combination [34].

## Funding

This work was carried out with financial support of the European Union's 7th Framework Programme FP7 2007–2013 under grant agreement n° 244995 (BIOCLAIMS Project) - recipients SK and PJT; Qatar University (Project code QU ERG-CMED-2020-1) - recipient NR; and Qatar Foundation (Project code QB-14) - recipient PJT.

## Intellectual property

We confirm that we have given due consideration to the protection of intellectual property associated with this work and that there are no impediments to publication, including the timing of publication, with respect to intellectual property. In so doing we confirm that we have followed the regulations of our institutions concerning intellectual property.

## Research ethics

This research did not involve human subjects. All animal experiments were performed in compliance with the German animal protection law (TierSchG).

## Author contributions

JM analysed skeletal muscle, plasma and urine samples, PW and NR analysed the proteomics data, SKe performed animal experiment and organ collection, MO, NR, PJT and SKl designed the study and checked all experimental data; and PJT and SKl drafted the manuscript. All authors checked, amended and approved the manuscript.

## Declaration of competing interest

The authors declare that they have no conflict of interest.

## Data Availability

Data will be made available on request.
