# Supplementary material for: Decreased methylglyoxal-mediated protein glycation in the healthy aging mouse model of ectopic expression of UCP1 in skeletal muscle
Source: Redox Biol. 2022 Dec 6;59:102574. doi: 10.1016/j.redox.2022.102574 (PMC9772855; doi:10.1016/j.redox.2022.102574)
Supplement: Multimedia component 1 [file mmc1.docx]

**Supplementary Tables**

**Table S1**. Protein glycation, oxidation and nitration adduct residues in skeletal muscle protein and young and aged WT and UCP1 transgenic mice.

| Protein damage marker | | Young | | Aged | | Significance  (P-value; ANOVA) |
| --- | --- | --- | --- | --- | --- | --- |
| Class | Marker | WT | HSA-mUCP1 | WT | HSA-mUCP1 |  |
| Glycation | FL (mmol/mol lys) | 0.117 ± 0.056 | 0.254 ± 0.042*** | 0.118 ± 0.025 | 0.201 ± 0.074*^,OO^ | <0.001 |
|  | CML (mmol/mol lys) | 0.090 ± 0.008 | 0.109 ± 0.021* | 0.120 ± 0.018* | 0.119 ± 0.017** | 0.003 |
|  | CEL (mmol/mol lys) | 0.0263 ± 0.0037 | 0.0196 ± 0.0046** | 0.0240 ± 0.0032 | 0.0159 ± 0.0076**^,OO^ | 0.002 |
|  | G-H1 (mmol/mol arg) | 0.0095 ± 0.0031 | 0.0130 ± 0.0067 | 0.0095 ± 0.0031 | 0.0114 ± 0.0027 |  |
|  | MG-H1 (mmol/mol arg) | 0.461 ± 0.135 | 0.476 ± 0.149 | 0.370 ± 0.052 | 0.268 ± 0.025**^,OOO,++^ | 0.003 |
|  | 3DG-H (mmol/mol arg) | 0.084 ± 0.031 | 0.118 ± 0.033* | 0.114 ± 0.008* | 0.116 ± 0.013* | 0.023 |
|  | CMA (mmol/mol arg) | 0.0243 ± 0.0038 | 0.0319 ± 0.0078* | 0.0216 ± 0.0046 | 0.0259 ± 0.0054 | 0.008 |
|  | PENT (mmol/mol lys) | 0.0136 ± 0.0025 | 0.0160 ± 0.0019* | 0.0104 ± 0.0023* | 0.0112 ± 0.0023^+++^ | <0.001 |
| Oxidation | AASA (mmol/mol lys) | 0.0177 ± 0.0067 | 0.0221 ± 0.0108 | 0.0206 ± 0.0073 | 0.0189 ± 0.0058 |  |
|  | GSA (mmol/mol lys) | <LOD | <LOD | <LOD | <LOD |  |
| Nitration | 3-NT (mmol/mol tyr) | 0.0044 ± 0.0022 | 0.0053 ± 0.0027 | 0.0032 ± 0.0017 | 0.0026 ± 0.0015^+^ |  |

Data are mean ± SD (n = 8). Significance: 4 group comparison – ANOVA (righthand column) and 2-group comparisons – Student’s t-test. Key: *, ** and ***, P<0.05, P<0.01 and P<0.001 with respect to young WT; oo and ooo, P<0.01 and P<0.001 with respect to aged WT; and +, ++ and +++, P<0.05, P<0.01 and P<0.001 with respect to young HSA-mUCP1 mice. Limit of detection (LOD) of GSA was <0.012 mmol/mol lys.

**Table S2**. Protein glycation, oxidation and nitration adduct residues in plasma protein and young and aged WT and UCP1 transgenic mice.

| Protein damage marker | | Young | | Aged | | Significance  (P-value; ANOVA) |
| --- | --- | --- | --- | --- | --- | --- |
| Class | Marker | WT | HSA-mUCP1 | WT | HSA-mUCP1 |  |
| Glycation | FL (mmol/mol lys) | 2.01 ± 0.27 | 1.91 ± 0.17 | 1.51 ± 0.32** | 1.41 ± 0.52*^,+^ | 0.006 |
|  | CML (mmol/mol lys) | 0.120 ± 0.015 | 0.110 ± 0.015 | 0.122 ± 0.020 | 0.128 ± 0.013^,+^ |  |
|  | CEL (mmol/mol lys) | 0.0159 (0.0110 – 0.0458) | 0.0163 (0.0088 – 0.0362) | 0.0093 (0.0057 – 0.0151) | 0.0171 (0.0135 – 0.0230) |  |
|  | G-H1 (mmol/mol arg) | 0.0154 ± 0.0042 | 0.0091 ± 0.0054* | 0.0118 ± 0.0062 | 0.0178 ± 0.0079^+^ |  |
|  | MG-H1 (mmol/mol arg) | 0.143 ± 0.036 | 0.145 ± 0.051 | 0.132 ± 0.028 | 0.135 ± 0.025 |  |
|  | 3DG-H (mmol/mol arg) | 0.097 ± 0.021 | 0.098 ± 0.035 | 0.074 ± 0.015* | 0.082 ± 0.014 |  |
|  | CMA (mmol/mol arg) | 0.0521 ± 0.0181 | 0.0432 ± 0.0098 | 0.0453 ± 0.0120 | 0.0521 ± 0.0141 |  |
|  | PENT (mmol/mol lys) | 0.0056 ± 0.0024 | 0.0046 ± 0.0027 | 0.0011 ± 0.0005** | 0.0005 ± 0.0004**^,++^ | <0.001 |
| Oxidation | AASA (mmol/mol lys) | 0.0125 ± 0.0086 | 0.0134 ± 0.0026 | 0.0052 ± 0.0015* | 0.0068 ± 0.0.0023^+++^ | 0.003 |
| Nitration | 3-NT (mmol/mol tyr) | 0.0242 ± 0.0097 | 0.0126 ± 0.0023* | 0.0192 ± 0.0077 | 0.0182 ± 0.0049^+^ |  |

Data are mean ± SD (n = 8). Significance: 4 group comparison – ANOVA (righthand column) and 2-group comparisons – Student’s t-test. Key: * and **, P<0.05 and P<0.01 with respect to young WT; and +, ++ and +++, P<0.05, P<0.01 and P<0.001 with respect to young HSA-mUCP1 mice. There was no significant difference between mean estimates of aged WT and aged HSA-mUCP1 for all analytes.

**Table S3. Urinary protein glycation free adduct excretion in aged WT and UCP1 transgenic mice.**

| Free adduct | Age | |
| --- | --- | --- |
|  | WT | HSA-mUCP1 |
| FL (nmol/mg creatinine) | 5.14 (2.17 – 11.27) | 4.44 (2.82 – 8.48) |
| CML (nmol/mg creatinine) | 0.92 (0.36 – 1.93) | 0.58 (0.50 – 0.73) |
| CML/FL (%) | 14.9 (12.6 – 22.9) | 12.8 (10.8 – 17.6) |
| CEL (nmol/mg creatinine) | 0.131 (0.061 – 0.405) | 0.103 (0.083 – 0.185) |
| G-H1 (nmol/mg creatinine) | 0.0073 (0.0034- 0.0182) | 0.0076 (0.0051 – 0.0120) |
| MG-H1 (nmol/mg creatinine) | 0.0438 (0.0209 – 0.0774) | 0.0252 (0.0190 – 0.0312) |
| (CEL+MG-H1)/FL (%) | 4.3 (3.4 – 4.6) | 1.7 (1.5 – 2.9)* |
| 3DG-H (nmol/mg creatinine) | 0.199 (0.109 – 0.564) | 0.163 (0.142 – 0.247) |
| CMA (nmol/mg creatinine) | 0.025 (0.015 – 0.114) | 0.025 (0.020 - 0.031) |
| PENT (nmol/mg creatinine) | 0.00061 (0.00023 – 0.00094) | 0.00070 (0.00046 – 0.00104) |

Data are median (lower – upper quartile; n = 10). Significance: *, P<0.05; Mann-Whitney U test.
